# Smoking among pregnant women in small towns in Poland

**DOI:** 10.1007/s00038-015-0735-2

**Published:** 2015-09-04

**Authors:** Łukasz Balwicki, M. Zarzeczna-Baran, Ł. Wierucki, T. Jędrzejczyk, M. Strahl, M. Wrotkowska, M. L. Goniewicz, T. Zdrojewski

**Affiliations:** Department of Public Health and Social Medicine, Medical University of Gdansk, Gdańsk, Poland; Department of Preventive Medicine and Education, Medical University of Gdansk, Gdańsk, Poland; Polish National Health Fund, Warsaw, Poland; Department of Obstetric and Gynaecological Nursing, Medical University of Gdansk, Gdańsk, Poland; Department of Hygiene and Epidemiology, Medical University of Gdansk, Gdańsk, Poland; Department of Heath Behavior, Roswell Park Cancer Institute, Buffalo, USA

**Keywords:** Smoking, Pregnancy, Smoking cessation, Epidemiology

## Abstract

**Objectives:**

The aim of the work was to assess among pregnant women from small towns and villages in Poland: the prevalence of smoking, credibility of smoking, and influence of socioeconomic factors on smoking status.

**Methods:**

The data came from 4512 interviews with women in different trimesters of pregnancy. The interviews were collected in 2007 and 2008 year in towns up to 8000 citizens in 12 voivodeships.

**Results:**

Prevalence of smoking in the beginning of pregnancy was confirmed by 34.6 % of women. During the pregnancy 14.7 % of women declared quitting smoking and 19.9 % continued smoking. Cigarette smoking was most frequent in those with a primary education, unemployed, very low incomes in household, and having both smoking parents. In multifactorial analysis, risk of smoking was highest (95 % CI 1.74–6.06) for women that were divorced or not living with a partner compared with married.

**Conclusions:**

Rates of active smoking among a population of pregnant women living in small towns in Poland are very high. Since the correlates of smoking during pregnancy are a low education level and a low economic status of the pregnant woman, these socioeconomic groups should be first priority targets.

## Introduction

Tobacco smoking represents a huge health hazard for both a pregnant woman and her fetus and it also represents the most important modifiable risk factor for fetal and neonatal morbidity and mortality (U.S. Department of Health and Human Services 2014). Pregnancy represents a conducive period when there is a valid incentive to quit smoking. Research into the incidence of active and passive smoking and the credibility of pregnant women’s declarations of not smoking will help document the scale of the problem in the population and help the public health community to design effective educational and intervention activities.

Poland is one of the countries in the region of Central and Eastern Europe (CEE) that went through a political and economic transformation in early 1990s. Health indicators in these countries were at that time much worse than the corresponding figures reported for the 15 ‘old’ members of the European Union. Despite an improvement in these indicators over the past two decades, the rate of changes has not been satisfactory (Helis et al. 2011). In Poland and other EU member states, significant health disparities have been reported between people living in small towns with higher unemployment rates and people living in large cities (Wojtyniak et al. 2012).

Since 2002, epidemiological studies carried out in Poland have indicated that, despite the introduction of various new legal restrictions and social education activities, no noticeable decline in the number of female smokers has been observed, even though the situation concerning smoking among the male population has improved (Jassem et al. 2014). The Polish edition of the Global Adult Tobacco Survey (GATS), conducted in Poland between November 2009 and March 2010, reported that on average in the population of adult females (i.e., over 20), 25 % smoke every day, 2 % from time to time, and 9 % are ex-smokers (Polish Ministry of Health 2012). The percentage of female smokers in the Polish population is still below that of male smokers (37 % of men smoke every day, 2 % from time to time, 20 % quit smoking, and 41 % have never smoked), but the gender gap has been decreasing over recent years (Chief Sanitary Inspectorate 2009).

A review of national surveys of tobacco smoking among women of reproductive age (Przewoźniak et al. 2009) has indicated that in Poland between 2000 and 2004, the prevalence of daily smoking was 35.8 % in large cities (over 100 K residents), 33.1 % in smaller towns, and 26 % in the countryside (Przewoźniak et al. 2009). However, none of these studies explored the smoking rates among pregnant women from disadvantaged populations. The objective of this study was to assess the prevalence of tobacco smoke exposure among pregnant women in small towns in Poland. We also aimed to evaluate the accuracy of self-reported smoking status among those women using validated biomarkers.

## Methods

### Sampling protocol

The study was a part of the national prevention program ‘Polish Project of 400 Cities’ run in Poland between 2007 and 2008. In this cross-sectional study, we recruited pregnant women using three-stage cluster sampling strategy. In the first stage, we ranked provinces based on unemployment rate, average per capita income, and level of social support. Twelve provinces with the worst indicators were included in the study. The provinces included: Kujawsko-Pomorskie, Lubelskie, Lubuskie, Łódzkie, Małopolskie, Opolskie, Podkarpackie, Podlaskie, Pomorskie, Świętokrzyskie, Warmińsko-Mazurskie and Zachodniopomorskie. In the second stage, we selected 179 small towns in each province. A town was considered as small if the number of residents did not exceed 8000 (Zdrojewski et al. 2006). In the third stage, in each selected city, we invited all gynecology and prenatal care clinics, as well as community midwife practices to participate in the study.

### Participants

We recruited 4512 pregnant women in the age range between 13 and 49 years. The total response rate was 94.6 %. All participants provided informed written consent. The study protocol was approved by the Bioethics Committee at the Medical University of Gdansk.

### Study protocol

Interviews were carried out between January 2007 and November 2008 in gynecology and prenatal care clinics or at community midwife practices. Interviews were carried out by specifically trained community midwives (see below). Midwives are usually considered by pregnant women as the group of healthcare professionals with whom a better emotional rapport can be established. They usually have more time for the patient and, being women themselves, show more empathy for another woman’s concerns regarding the course of pregnancy. These factors lead to a greater trust being placed in them, which was a factor is contributing to truthful reporting of facts in a survey where the respondent was not completely anonymous at the time of data collection. This lack of anonymity resulted from the fact that the questionnaire was administered during a routine visit at a gynecology and prenatal care clinic. Anonymization of data was carried out only after the visit.

Prior to the research roll-out, the midwives had been specifically trained in interviewing methods and the use of carbon monoxide meters as described below. The interview was conducted once with all pregnant women who reported for their first or subsequent consultation with a midwife, regardless of the stage of pregnancy, within the 3 months of program implementation in each province.

### Research tool (questionnaire)

The questionnaire, developed specifically for the purposes of this study, consisted of 34 questions, of which 24 were about active and passive tobacco smoking, and 10 were about socioeconomic factors. Following the approach used by Mullen et al., a question concerning regular daily smoking before and during pregnancy was used for initial screening (Mullen et al. 1991). Because smoking in pregnancy is a socially unaccepted behavior, women are reluctant to disclose the truth of their active smoking status, and this may potentially lead to a considerable gap between the self-reported and actual smoking status, as measured by biochemical markers (Fendrich et al. 2005; Pérez-Stable et al. 1990; Secker-Walker et al. 1997; Ford et al. 1997). From the viewpoint of medical practice, the consequence of misreported status may be that a doctor or nurse will not be able to provide proper care if they are not aware of the actual exposure to tobacco smoke (Russell et al. 2004; Connor Gorber et al. 2009). Being aware that there may be a high incidence of unreported smoking levels and aiming at significantly reducing misclassification error confirmed with biochemical tests in earlier surveys, we used a screening question which provided respondents with more answers than a straightforward ‘yes’ or ‘no’ (Cummings et al. 1990; Pérez-Stable et al. 1992). Respondents had several options to best describe their behavior concerning smoking in general, and smoking/quitting in pregnancy. Nine questions were addressed only to the women who continued smoking during pregnancy, and four to those who ceased smoking before or during pregnancy.

### Biochemical verification of smoking status

We decided to use a biochemical marker of cigarette smoking, such as exhaled carbon monoxide to verify self-reported smoking status (Florek et al. 2004a). Following the interview, those patients who had given consent were tested with a meter measuring carbon monoxide in exhaled air (MicroCO, MicroMedical, UK). According to the manual provided by manufacturer, a result of >7 ppm CO in exhaled air indicates current smoking and this cutoff value was used in this study. The CO breath test was carried out in 4145 pregnant women; 98 women refused the test and 269 were not tested because the device was not available or was not working properly in the time of testing. Overall among 3587 women who self-reported non-smoking status at the time of the test, 6.0 % were not confirmed as a non-smoker by a carbon monoxide meter test (CO threshold ≥7 ppm), and another 2.2 % refused the test.

### Statistical analysis

The interview database was statistically processed and analyzed for correlations. Categorical variables were analyzed according to frequency reports. In the processing of data descriptive statistics were used. To determine correlations between the smoking status and socioeconomic factors, the Chi-square test, unifactorial and multifactorial logistic regression were used. Multifactorial logistic regression included the correction for the effect of age and the potential impact of other factors on the tested relationship. Only variables that were significant with a *p* value <0.2 in the unifactorial analysis were included in the multifactorial analysis. The significance level was ≤0.05. The software used for statistical analyses was Statistica 9.1 and STATA 9.

## Results

### Characteristics of study population

The research population consisted of pregnant women in the age range between 13 and 49 years. The basic demographic parameters of the population are shown in Table 1.

**Table 1:** Characteristics of the pregnant women in Poland surveyed between 2007 and 2008 (*N* = 4512)

Variable	Mean ± SD or %
Age (years)	26.9 ± 5.6
Stage of pregnancy at the time of test (weeks)	24.7 ± 9.8
Trimester	
First	16.9 %
Second	36.5 %
Third	46.4 %
Missing data	0.2 %
Which pregnancy	
First	43.6 %
Second or more	54.8 %
Missing data	1.6 %
Education level	
Primary school	16.0 %
Post-primary vocational education	28.2 %
Secondary education (high school)	41.3 %
University-level education	13.3 %
Missing data	1.2 %
Marital status	
Married	72.8 %
Informal relationship	15.1 %
Single	10.9 %
Missing data	1.3 %
Education level of spouse/partner	
Primary	11.9 %
Post-primary vocational	48.6 %
Secondary (high school)	29.1 %
University	8.0 %
Missing data	2.4 %
Employment status	
Student	9.2 %
Employed	39.8 %
Unemployed	30.4 %
Running/managing the family’s home	16.9 %
None of the above	2.4 %
Missing data	1.2 %
Net per capita income	
Very low (<350 PLN)	31.5 %
Low (350–650 PLN)	37.9 %
Average (651–1000 PLN)	18.0 %
High (>1000 PLN)	9.7 %
Missing data	3.0 %
Smoking status of parents/guardians	
None	19.9 %
Only father	34.5 %
Only mother	8.6 %
Both	35.5 %
Missing data	1.5 %

### Exposure to tobacco smoke among pregnant women living in small towns in Poland

**Fig. 1:**
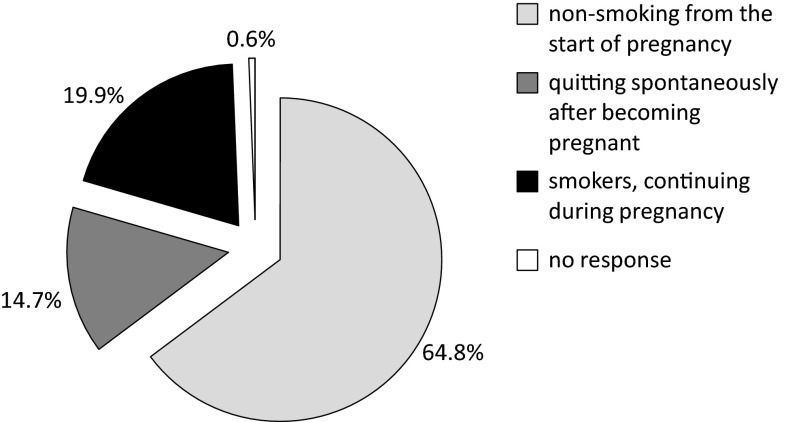
Pregnant women in Poland according to their self-reported smoking status in 2007–2008 (*N* = 4512)

We found that one in five women in the study continued smoking during their pregnancy (being aware of the pregnancy) (Fig. 1). With the nearly 15 % of women who claimed to have spontaneously quit smoking after becoming pregnant, we have almost 35 % of the research population who admitted to smoking at the onset of pregnancy. This also means that only 42.5 % of female smokers participating in the survey declared to quit the habit because of their pregnancy. Should all the pregnant women who admitted to active smoking (19.9 %) and all those for whom the CO test count was at least 7 ppm (4.8 % of the entire tested population) be classified as active smokers, the resulting proportion would be 24.7 %. Moreover, 80 self-reported non-smokers (another 1.8 % of the entire population) refused to take part in the survey, and these women may also be counted as likely tobacco smokers.

**Fig. 2:**
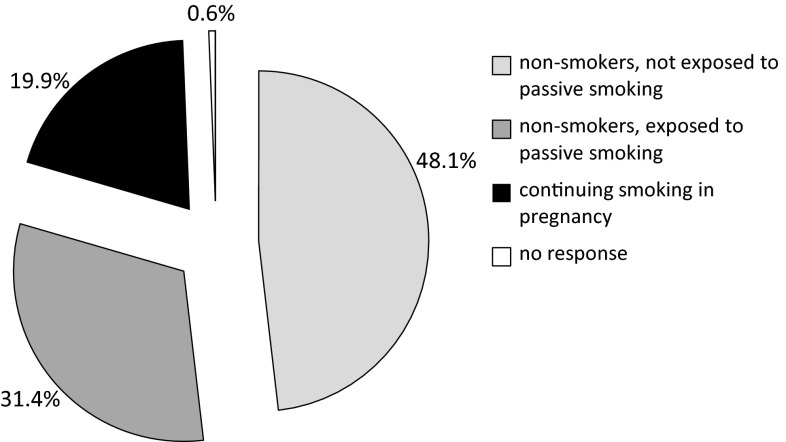
Summary exposure to active and passive smoking among pregnant women in Poland in 2007–2008 (*N* = 4512)

To deliver a comprehensive assessment of the exposure of pregnant women to tobacco smoke, the percentages of those who continue smoking and of non-smokers who are exposed to passive smoking have also been considered together (Fig. 2). Taking into account both active and passive tobacco smoke exposure, 51.3 % of pregnant women were exposed to tobacco smoke.

### Biochemical verification of the self-reported smoking status

Among 664 the women who declared being ex-smokers and who spontaneously quit at the onset of pregnancy, the 15.5 % misreported their non-smoking status and 3.5 % refused to be tested.

### Socioeconomic correlates of smoking in pregnancy

The relationship between smoking in pregnancy and selected socioeconomic factors is shown in Table 2. The highest rate of smokers who do not quit in pregnancy was found among women with primary school education, those in an informal relationship, with the lowest per capita income, and whose parents were both smokers. The odds ratios (OR) were calculated for each factor and presented in Table 2.

**Table 2:** Risk factors of continuing smoking during pregnancy among pregnant Polish women living in small towns (data from 2007–2008)

Variables	Multifactorial	Unifactorial
	AOR (95 % CI)	OR (95 % CI)
Age	1.02 (1.00–1.03)	1.04 (1.03–1.06)
Education level		
Primary	Ref	Ref
Post-primary vocational	0.68 (0.55–0.84)	0.46 (0.37–0.55)
Secondary (high school)	0.43 (0.35–0.53)	0.22 (0.18–0.27)
University	0.26 (0.18–0.36)	0.05 (0.03–0.08)
Marital status		
Married	Ref	Ref
Informal relationship	2.46 (2.03–2.99)	3.47 (2.89–6.83)
Divorced or not living with a partner	3.25 (1.74–6.06)	4.09 (2.45–6.83)
Single and not living with a partner	1.71 (1.32–2.21)	2.71 (2.17–3.40)
Education level of spouse/partner		
Primary	Ref	Ref
Post-primary vocational	1.10 (0.88–1.37)	0.70 (0.57–0.86)
Secondary (high school)	1.01 (0.79–1.30)	0.38 (0.30–0.48)
University	0.65 (0.43–0.97)	0.11 (0.07–0.19)
Employment status		
Student	Ref	Ref
Employed	0.96 (0.72–1.28)	0.64 (0.49–0.83)
Unemployed	1.16 (0.89–1.52)	1.59 (1.22–2.07)
Running/managing family’s home	0.72 (0.52–0.99)	0.79 (0.59–1.07)
Net per capita income		
Very low (<350 PLN)	Ref	Ref
Low (350–650 PLN)	1.17 (0.99–1.39)	0.62 (0.53–0.74)
Average (651–1000 PLN)	1.10 (0.88–1.37)	0.49 (0.39–0.61)
High (>1000 PLN)	1.03 (0.73–1.46)	0.34 (0.23–0.49)
Smoking status of parents/guardians		
None	Ref	Ref
Only father	1.51 (1.22–1.88)	1.37 (1.12–1.68)
Only mother	2.13 (1.60–2.85)	2.53 (1.94–2.31)
Both	3.26 (2.64–4.02)	4.34 (3.57–5.27)

With regard to active smoking, the most significant variables were ‘divorced or not living with a partner’ (AOR 3.25; 95 % CI 1.74–6.06) and ‘both parents were smokers’ (AOR 3.26; 95 % CI 2.64–4.02). Other statistically significant factors in the multifactorial analysis included: lower education status of the pregnant woman, lower education status education of the spouse/partner, social status of the pregnant woman (unemployed), and the age of the pregnant woman. In unifactorial analysis, the same variables were found to be significant predictors (OR) of smoking in pregnancy.

## Discussion

We provided novel data on smoking prevalence among pregnant women in small towns in Poland. Early population-based studies included the entire group of women of childbearing age which, consequently, makes the interpretation of results at a very detailed level of small town population rather difficult. Our data showed that at the time when the survey was taken, over one in three women were smoking tobacco cigarettes at the beginning of their pregnancy. This number is higher than 24.4 % national overall/average smoking prevalence among women reported in 2009 in the GATS study (aged 15–19: 12.1 %, 20–29: 27.2 %, 30–39: 25.8 %; living in rural areas: 20.2 % vs. in cities: 26.8 %. The number is also higher than the 21.9 % smoking rate (aged 18–24: 11.7 % and 25–34: 21.4 %) reported by pregnant women in Lodz, one of the poorest cities in Poland (Fronczak et al. 2012). One study found that although 25.0 % of pregnant women in Poland smoked 3 months before conception, the prevalence of daily smoking decreased to 12.0 % in third trimester (Wojtyła et al. 2012).

When the rates of smokers in pregnancy in our survey are compared with studies carried out in other countries, we can see a quite wide range of results. In Western and Northern Europe, the rates are: Finland—14 %, Germany—15 %, France—18 %, United Kingdom—27 %; in Eastern Europe: Croatia—18.9 %, Serbia—18.4 %, Russia—9.7 %, Slovenia: 6.7 %; and in the United States—13 % (Smedberg et al. 2014; Schneider and Schütz 2008; Schneider et al. 2008). These differences in the incidence of smoking are not easy to interpret because they may be associated with a variety of factors, including the general prevalence of smoking among women of reproductive age in specific countries, awareness levels among women who are getting pregnant, the quality of antenatal care, different population strategies adopted in specific countries to fight the tobacco epidemic or, finally, methodological differences between individual studies. Many high-income countries report declining rates of smoking during pregnancy over the past 20 years (Meernik and Goldstein 2015).

An important finding of this study is that majority of smoking women in small towns in Poland continue to smoke during pregnancy. Future studies need to explore the barriers towards quitting that smoking women who live in rural area in Poland may experience. Polańska et al. addressed the lack of studies to analyze the prevalence of smoking at different stages into pregnancy (Polańska et al. 2007). In the study conducted in prenatal care clinics in the city of Łódź, the proportion of pregnant women who admitted to smoking cigarettes ranged between 25 and 30 % depending on the trimester.

Although an interview-based survey is the least expensive method of assessing tobacco smoke exposure, and the survey results are immediately ready for evaluation, there are possible sources of bias when smoking status information is based on self-report only, such as respondents forgetting about relevant facts, their unwillingness to disclose the information sought by researchers, and intentionally providing misleading information (Solberg 1996). It has been shown previously that social pressure on pregnant smokers causes some of them misreport their status as non-smokers, contrary to facts (Campbell et al. 2001; Lapham et al. 1991; Hughes et al. 1982; Wagenknecht et al. 1992). The results of this study show a relatively high consistency of self-reported smoking status with the facts and a rather high degree of truthful reporting. The exception to this was the group of pregnant women who declared they have quit smoking. This is consistent with findings of other studies; George et al. indicated about 13 % of misreported non-smoking in a group who declared to have ceased smoking before the interview (George et al. 2006). In our survey, this proportion was even higher (almost 16 %). In earlier Polish studies in which cotinine levels in urine were used as a biomarker, the level of unreported smoking was as high as 15.5 % at 50 ng cotinine/mg creatinine cutoff point (Florek et al. 2004b). In our survey, about 6 % of pregnant women who declared they did not smoke had an increased level of exhaled carbon monoxide, which provided an indication of active smoking. This translates into 4.8 % of the entire population, and brings the actual rate of smoking in pregnancy to 25 %. In the research by Klebanoff et al. in which the accuracy of self-reporting of pregnant women’s smoking status in 1960s and 1990s was compared, the recent biomarker data were virtually identical, as to their accuracy, to those obtained in a pregnancy cohort from the 1960s. The cohort study confirmed self-reported status of 84.6 % smokers and 94.5 % of women who denied smoking; the misreporting rates seem to not have changed substantially, despite the fact that pressure on pregnant smokers to quit has increased over the past decades (Klebanoff et al. 2001).

Although there is strong evidence that tobacco smoking is highly associated with lower socioeconomic status (Escobedo et al. 1995; CSDH 2008; Meernik and Goldstein 2015), little is known about smoking rates among pregnant women from disadvantaged populations. Dejin-Karlsson et al. indicated the factors contributing to smoking in pregnancy to be low socioeconomic status, an unplanned pregnancy, a lack of support from the partner, and stress in the workplace. Other characteristic risk factors recognized by the authors were young age, lower educational status, hard physical work (Dejin-Karlsson et al. 1996). Polańska et al. demonstrated that smoking rates in Poland were significantly higher among unmarried (60 %) compared to married women (30 %); or those with primary or post-primary vocational education (70 %) compared to those with secondary education (45 %) (Polańska et al. 2007). The results of our study confirmed the relationship between the incidence of smoking and education level. The higher the educational level of pregnant women, the lower the proportion of those who continue smoking in pregnancy. Among women with university-level education, only 2.7 % admitted to smoking in pregnancy, compared to 42.2 % among those with primary education. Marital status is another variable where there was a clear correlation with smoking in pregnancy: significantly fewer women who were married continued smoking in pregnancy when compared to women in an informal relationship and those who were single or not living with a partner. These findings are consistent with other studies, suggesting that marriage provides a higher sense of security and better social support, which results in fewer risky behaviors and, consequently, better health (Schoenborn 2004). Our study also indicated a relationship between smoking by pregnant women and the smoking habit of their parent(s)/guardian(s). Having smoking parents significantly increased the odds of smoking during pregnancy. This is consistent with earlier reports showing the strong association between children and parent smoking status (Farkas et al. 2000; Proescholdbell et al. 2000; Kelly et al. 2011; de Vries et al. 2003; Meernik and Goldstein 2015).

### Conclusions

Rates of active smoking among population of pregnant women living in small towns in Poland are very high. Our study indicates an urgent need for public health interventions aimed at reducing the incidence of smoking during pregnancy in this disadvantaged population. Effective methods of reducing the tobacco epidemic at both the individual and population levels, including smoking cessation treatment and counseling are available in Poland; however, the access to these resources in small towns may be significantly limited. Since the correlates of smoking during pregnancy are a low education level and a low economic status of the pregnant woman, these are the socioeconomic groups that should be targeted as the first priority. Poland is a country with a high level of socioeconomic inequalities and there is a need to close these gaps.

The fact that a considerable proportion of women spontaneously quit smoking after being found pregnant indicates that pregnancy is a strong motivating factor behind the cessation of smoking. This is why it is worthwhile to use the time of pregnancy as a period in which interventions should be applied.
